# Supplementary material for: Changing course: Glucose starvation drives nuclear accumulation of Hexokinase 2 in S. cerevisiae
Source: PLoS Genet. 2023 May 17;19(5):e1010745. doi: 10.1371/journal.pgen.1010745 (PMC10228819; doi:10.1371/journal.pgen.1010745)
Supplement: S1 Text — (DOCX) [file pgen.1010745.s001.docx]

**Supporting Information**

**Changing course: Glucose starvation drives nuclear accumulation of Hexokinase 2 in *S. cerevisiae***

Mitchell A. Lesko^1^, Dakshayini G. Chandrashekarappa^2^, Eric Jordahl^1^, Katherine G.

Oppenheimer^1^, Ray Wesley Bowman^1^, Chaowei Shang^1^, Jacob Durrant^1^, Martin C.

Schmidt^2^*, and Allyson F. O’Donnell^1^*

**Supplemental Text: Results & Materials and Methods**

**Supplemental Results**

**Molecular dynamics simulations suggest glucose binding promotes monomerization**

To explore potential glucose/N-terminal-tail antagonism, we performed molecular dynamics simulations of three systems based on our homology model: (1) the *holo* (glucose-bound) *Sc*Hxk2 monomer, (2) the *apo* (ligand-free) *Sc*Hxk2 dimer, and (3) the *holo* (glucose-bound) *Sc*Hxk2 dimer. We did not include ATP in these simulations because we hoped to capture the impact of the initial glucose-binding event before ATP binding and glucose phosphorylation. Simulations of the *holo* (glucose-bound) monomer served to rule out the possibility that starting the simulations in an open conformation alone would allow for glucose dissociation, even when an N-terminal tail does not occupy the cleft. We performed three independent simulations of the *holo* monomer starting from the open (dimer-like) conformation (1,043 ns total). At no point did the glucose molecule deviate substantially from its initial binding pose (mean RMSD: 2.24 Å; standard deviation: 0.89 Å).

Simulations of the *apo* (glucose-absent) dimer model served to rule out the possibility that the N-terminal tail is prone to dissociate independently of bound glucose, perhaps due to inaccuracies in our homology model. We performed three simulations of the *apo* *Sc*Hxk2 dimer (1,078 ns total). At no point did any N-terminal tail dissociate from its opposite-monomer cleft.

In contrast, simulations of the *holo* (glucose-bound) *Sc*Hxk2 dimer captured notable glucose and N-terminal-tail dynamics. We performed three simulations of this system. In the first (262 ns total), both N-terminal tails remained associated with their respective opposite-monomer enzymatic clefts. The pose of one glucose molecule also remained stable (mean RMSD: 1.04 Å; standard deviation: 0.25 Å), but the other glucose was highly mobile within the cleft (mean RMSD: 5.11 Å; standard deviation: 2.56 Å). The second simulation of the *holo* *Sc*Hxk2 dimer (261 ns total) was similar. Both N-terminal tails remained associated with their respective clefts, one glucose remained stable (mean RMSD: 2.45 Å; standard deviation: 0.87 Å), and one glucose was mobile within its cleft (mean RMSD: 5.93 Å; standard deviation: 3.25 Å). Given that we saw little glucose mobility in 1,043 ns of *holo* *Sc*Hxk2 monomer simulation, we hypothesize that dimerization—and perhaps the opposite-monomer N-terminal tail specifically—discourages stable glucose binding at the enzymatic site.

The third simulation of the *holo* *Sc*Hxk2 dimer (1,000 ns total) explored dimer dynamics on slightly longer timescales. During this simulation, one of the N-terminal tails remained associated with its respective opposite-monomer enzymatic cleft. The glucose molecule in the same cleft was again highly mobile, so much so that it dissociated entirely from the protein. In contrast, the other N-terminal tail dissociated from its enzymatic cleft. The same-cleft glucose molecule was also highly mobile, though it remained in the cleft (average RMSD: 9.60 Å; standard deviation: 2.52 Å). Given that we saw little N-terminal-tail mobility in 1,078 ns of *apo* Hxk2 dimer simulation, we hypothesize that bound glucose encourages N-terminal-tail dissociation.

### **Proposed mechanism of initial dimer dissociation**

Because the third simulation captured the only observed tail dissociation, we explored its mechanistic details in greater depth. Early in the simulation, the positively charged terminal amine group of V2 is sandwiched between D458* and D417*. Other nearby inter-monomer interactions include the K7-E457* salt bridge and the Q10-T107*, Q10-D106*, and Q10-F105* hydrogen bonds (S5A Fig).

A dissociation cascade begins when the bound glucose molecule transiently reorients to form hydrophobic contacts with the V2 sidechain (S5B Fig). This brief interaction has two consequences. First, it expels the glucose molecule to a cleft-bound position distant from the N-terminal tail. Second, it weakens the V2-D458* interaction such that V2 interacts more exclusively with D417*. This change permits a new hydrogen bond to form between H3 and D86*, possibly partially compensating for the weakened V2-D458* interaction (S5C Fig).

However, the V2-D417* and H3-D86* interactions break after ~130 ns, causing the N-terminal tail to move away from the cleft. This large-scale movement impacts other inter-monomer interactions as well. Within about 100 ns, the K7-E457* interaction breaks, permitting the formation of a new K7-D417* interaction. Q10 also prefers Q109* and D458* as hydrogen-bond partners rather than T107*, D106*, and F105* as before. Amino acid K8, which did not previously interact with the opposite monomer, forms a strained electrostatic interaction with E457* (S5D Fig).

Two subsequent conformational rearrangements further destabilize the N-terminal-tail/cleft association. The first rearrangement occurs after another ~230 ns. H3, now free of D86*, forms a cation-π interaction with R423*, and Q10 returns to its hydrogen bond with T107* (S5E Fig). The second rearrangement occurs after another ~270 ns when V2 forms a salt bridge with E352* that further distances it from the cleft. The H3-D86* cation-π interaction breaks to accommodate this change, and H3 instead forms a hydrogen bond with the backbone carbonyl oxygen atom of N422* (S5F Fig).

These dynamics support the hypothesis that an N-terminal tail and glucose molecule do not simultaneously occupy the same enzymatic cleft. Of note, neither of the two *Kl*Hxk1-dimer crystal structures that fully resolve the N-terminal tail (PDB IDs 3O1W and 3O4W [1]) includes a bound glucose molecule. The one dimer structure that does include a glucose molecule (PDB ID 3O5B) does not have a fully resolved N-terminal tail, perhaps because the tail cannot bind (and be stabilized by) the opposite-monomer enzymatic cleft.

Our simulations captured only one N-terminal-tail dissociation event, so we cannot guarantee that this dissociation pathway is the most common. We further note that the simulation did not run long enough to capture the full dissociation of the entire dimer, which may occur on far longer (perhaps computationally intractable) timescales.

**Supplemental Materials and Methods**

### **Molecular dynamics simulations**

We performed molecular dynamics (MD) simulations of the *apo* dimer, *holo* dimer, and *holo* monomer systems. In each case, we used *tleap* (Ambertools18 [2]) to add a water box extending 10 Å beyond the protein along all dimensions. We also added Na+ counter ions sufficient to achieve electrical neutrality and then additional Na+ and Cl- counter ions to approximate a 150 mM solution. The protein, counter ions, water molecules, and glucose molecules were parameterized per the Amber ff14SB [3], TIP3P [4], and GLYCAM_06j-1 [2] force fields, respectively.

We applied four rounds of minimization using the Amber MD engine [5,6]. First, we minimized all hydrogen atoms for 5,000 steps. Second, we minimized all hydrogen atoms and water molecules for 5,000 steps. Third, we minimized all hydrogen atoms, water molecules, and protein side chains for 5,000 steps. Finally, we minimized all atoms for 10,000 steps.

After minimization, we equilibrated each system using three rounds of simulation. First, we subjected each system to a short simulation in the canonical ensemble (NVT, 0.02 ns total), with a 1.0 kcal/mol/Å^2^ restraining force applied to the backbone atoms. Using the same backbone restraints, we continued the simulation in the isothermal–isobaric ensemble (NPT, 1 atm, 1.0 ns total). Finally, we finished the equilibration (NPT, 1 atm, 1.0 ns total) without restraints. In all cases, we used a 2-fs timestep and a 310 K temperature setting.

After minimization and equilibration, we ran three isothermal-isobaric (NPT, 310 K, 1 atm) productive simulations of the *Sc*Hxk2 *apo* dimer (550 ns, 262 ns, 266 ns), the *Sc*Hxk2 *holo* dimer (1000 ns, 261 ns, 262 ns), and the *Sc*Hxk2 *holo* monomer (530 ns, 253 ns, 260 ns). We used a different random seed for each simulation.

### **RMS and pairwise distance analyses**

To calculate how far a bound glucose molecule deviated from its initial position, we used VMD [7] to align the associated *Sc*Hxk2 monomer by its alpha carbons. We then calculated the heavy-atom root mean square distances (RMSDs) between the starting glucose pose and the pose of each frame.

To monitor the hydrogen bond between K13 and Q142*, we used VMD to calculate the distance between the K13 terminal nitrogen atom and the Q142* sidechain carbonyl oxygen atom. We assumed a hydrogen bond had formed when this distance was less than 4.0 Å. To monitor the salt bridge between K13 and D106*, we calculated the distance between the K13 terminal nitrogen atom and the D106* terminal-most carbon atom. We assumed a salt bridge had formed when this distance was less than 4.0 Å.

### **Molecular visualization**

Images were generated using VMD [7] and BlendMol [8].

**Supplemental References**

1. Kuettner EB, Kettner K, Keim A, Svergun DI, Volke D, Singer D, et al. Crystal structure of hexokinase KlHxk1 of kluyveromyces lactis: A molecular basis for understanding the control of yeast hexokinase functions via covalent modification and oligomerization. Journal of Biological Chemistry. 2010;285: 41019–41033. doi:10.1074/jbc.M110.185850

2. Kirschner KN, Yongye AB, Tschampel SM, González-Outeiriño J, Daniels CR, Foley BL, et al. GLYCAM06: A generalizable biomolecular force field. Carbohydrates. J Comput Chem. 2008;29: 622–655. doi:10.1002/JCC.20820

3. Maier JA, Martinez C, Kasavajhala K, Wickstrom L, Hauser KE, Simmerling C. ff14SB: Improving the Accuracy of Protein Side Chain and Backbone Parameters from ff99SB. J Chem Theory Comput. 2015;11: 3696–3713. doi:10.1021/ACS.JCTC.5B00255

4. Jorgensen WL, Chandrasekhar J, Madura JD, Impey RW, Klein ML. Comparison of simple potential functions for simulating liquid water. J Chem Phys. 1998;79: 926. doi:10.1063/1.445869

5. Case DA, Cheatham TE, Darden T, Gohlke H, Luo R, Merz KM, et al. The Amber biomolecular simulation programs. J Comput Chem. 2005;26: 1668–1688. doi:10.1002/JCC.20290

6. Salomon-Ferrer R, Case DA, Walker RC. An overview of the Amber biomolecular simulation package. Wiley Interdiscip Rev Comput Mol Sci. 2013;3: 198–210. doi:10.1002/WCMS.1121

7. Humphrey W, Dalke A, Schulten K. VMD: Visual molecular dynamics. J Mol Graph. 1996;14: 33–38. doi:10.1016/0263-7855(96)00018-5

8. Durrant JD. BlendMol: advanced macromolecular visualization in Blender. Bioinformatics. 2019;35: 2323–2325. doi:10.1093/BIOINFORMATICS/BTY968
