# Supplementary material for: Changing course: Glucose starvation drives nuclear accumulation of Hexokinase 2 in S. cerevisiae
Source: PLoS Genet. 2023 May 17;19(5):e1010745. doi: 10.1371/journal.pgen.1010745 (PMC10228819; doi:10.1371/journal.pgen.1010745)
Supplement: S1 Table — (DOCX) [file pgen.1010745.s013.docx]

**S1 Table.** Yeast strains

| **Strain** | **Genotype** | **Source** |
| --- | --- | --- |
| BY4742 | *MAT* α *his3Δ1 leu2Δ0 lys2Δ0 ura3Δ0* | [1] |
| MSY1212 | *MAT* **a** *ura3-52 leu2Δ1 his3Δ200* | [2] |
| AFO3935 | *MAT* α *ura3Δ0 leu2Δ0 his3Δ1 hxk2Δ::KANMX4 TPA1-mScarlet::HYGRO* | This study.  Using the Tpa1-mScarlet strain from the mScarlet C-SWAT collection [3], we PCR amplified the mScarlet::HYGRO cassette using primers with homology to the C-terminal and downstream regions of the Tpa1 coding sequence. We transformed the Tpa1-mScarlet::HYGRO cassette into MSY1254 [4]. We then validated that the mScarlet::HYGRO cassette was integrated next to Tpa1 using a PCR-based approach. |
| AFO4345 | *MAT* α *ura3Δ0 leu2Δ0 his3Δ1 hxk2Δ::KANMX4 tda1Δ::NAT TPA1-mScarlet::HYGRO* | This study.  Using the Tpa1-mScarlet strain from the mScarlet C-SWAT collection [23], we PCR amplified the mScarlet::HYGRO cassette using primers with homology to the regions upstream and downstream of the Tpa1 coding sequence. Using pBB1 as a template, we PCR amplified the NAT cassette using primers with homology to the regions upstream and downstream of the Tda1 coding sequence. We transformed the Tpa1-mScarlet::HYGRO cassette into MSY1254 [4]. We then validated that the mScarlet::HYGRO cassette was integrated next to Tpa1 and the NAT drug resistance marker replaced the endogenous *TDA1* gene using a PCR-based approach. |
| AFO4354 | *MAT* α *ura3Δ0 leu2Δ0 his3*  *Δ1 met15Δ0 hxk1Δ::KAN TPA1-mScarlet::HYGRO* | This study.  Using the Tpa1-mScarlet strain from the mScarlet C-SWAT collection [3], we PCR amplified the mScarlet::HYGRO cassette using primers with homology to the C-terminal and downstream regions of the Tpa1 coding sequence. We transformed the Tpa1-mScarlet::HYGRO cassette into RG5867 [4]. We then validated that the mScarlet::HYGRO cassette was integrated next to Tpa1 using a PCR-based approach. |
| AFO4547 | *MAT* α *ura3Δ0 leu2Δ0 his3Δ1 glk1Δ::KAN TPA1-mScarlet::HYGRO* | This study.  Using the Tpa1-mScarlet strain from the mScarlet C-SWAT collection [3], we PCR amplified the mScarlet::HYGRO cassette using primers with homology to the C-terminal and downstream regions of the Tpa1 coding sequence. We transformed the Tpa1-mScarlet::HYGRO cassette into MSY1471 [4]. We then validated that the mScarlet::HYGRO cassette was integrated next to Tpa1 using a PCR-based approach. |
| MSY1475 | *MAT* α *ura3∆0 leu2∆0 his3∆1 met15∆0 hxk1∆::KAN hxk2∆::KAN glk1∆::KAN* | [4] |
| AFO3936 | *MAT* α *ura3∆0 leu2∆0 his3∆1 met15∆0 hxk1∆::KAN hxk2∆::KAN glk1∆::KAN TPA1-mScarlet::HYGRO* | This study.  Using the Tpa1-mScarlet strain from the mScarlet C-SWAT collection [3], we PCR amplified the mScarlet::HYGRO cassette using primers with homology to the C-terminal and downstream regions of the Tpa1 coding sequence. We transformed the Tpa1-mScarlet::HYGRO cassette into MSY1475 [4]. We then validated that the mScarlet::HYGRO cassette was integrated next to Tpa1 using a PCR-based approach. |
| AFO4705 | *MAT* α *ura3Δ0 leu2Δ0 his3Δ1 lys2Δ0 hxk2∆::KAN snf1Δ10 TPA1-mScarlet::HYGRO* | This study.  Using the Tpa1-mScarlet strain from the mScarlet C-SWAT collection [3], we PCR amplified the mScarlet::HYGRO cassette using primers with homology to the C-terminal and downstream regions of the Tpa1 coding sequence. We transformed the Tpa1-mScarlet::HYGRO cassette into MSY1261 (this study). We then validated that the mScarlet::HYGRO cassette was integrated next to Tpa1 using a PCR-based approach. |
| AFO4707 | *MAT* α *ura3Δ0 leu2Δ0 his3Δ1 hxk2Δ::KAN mig1Δ::KAN TPA1-mScarlet::HYGRO* | This study.  Using the Tpa1-mScarlet strain from the mScarlet C-SWAT collection [3], we PCR amplified the mScarlet::HYGRO cassette using primers with homology to the C-terminal and downstream regions of the Tpa1 coding sequence. We transformed the Tpa1-mScarlet::HYGRO cassette into MSY1590 (this study). We then validated that the mScarlet::HYGRO cassette was integrated next to Tpa1 using a PCR-based approach. |
| AFO4748 | *MAT* α *ura3-52 leu2Δ1 his3Δ200 reg1Δ::HIS3 TPA1-mScarlet::HYGRO* | This study.  Using the Tpa1-mScarlet strain from the mScarlet C-SWAT collection [3], we PCR amplified the mScarlet::HYGRO cassette using primers with homology to the C-terminal and downstream regions of the Tpa1 coding sequence. We transformed the Tpa1-mScarlet::HYGRO cassette into MSY1226 (this study). We then validated that the mScarlet::HYGRO cassette was integrated next to Tpa1 using a PCR-based approach. |
| Tda1-mNG strain | *MAT a his3Δ1 leu2Δ0 met15Δ0 ura3Δ0 Tda1-mNeonGreen* | [3] |
| AFO4545 | *MAT a his3Δ1 leu2Δ0 met15Δ0 ura3Δ0 Tda1-mNeonGreen Tpa1-mScarlet::HYGRO* | This study.  Using the Tpa1-mScarlet strain from the mScarlet C-SWAT collection [3], we PCR amplified the mScarlet::HYGRO cassette using primers with homology to the C-terminal and downstream regions of the Tpa1 coding sequence. We transformed the Tpa1-mScarlet::HYGRO cassette into the Tda1-mNG strain. We then validated that the mScarlet::HYGRO cassette was integrated next to Tpa1 using a PCR-based approach. |
| YSH202  (W303 background) | *MAT****a*** *ura3-1 leu2-3/112 trp1-1 his3-11/15 ade2-1 can1-100* | [5] |

**S1 Table References**

1. Baker Brachmann C, Davies A, Cost GJ, Caputo E, Li J, Hieter P, et al. Designer Deletion Strains derived from Saccharomyces cerevisiae S288C: a Useful set of Strains and Plasmids for PCR-mediated Gene Disruption and Other Applications. Yeast. 1998;14: 115–132. doi:10.1002/(SICI)1097-0061(19980130)14:2

2. McCartney RR, Chandrashekarappa DG, Zhang BB, Schmidt MC. Genetic analysis of resistance and sensitivity to 2-deoxyglucose in Saccharomyces cerevisiae. Genetics. 2014;198: 635–646. doi:10.1534/genetics.114.169060

3. Meurer M, Duan Y, Sass E, Kats I, Herbst K, Buchmuller BC, et al. Genome-wide C-SWAT library for high-throughput yeast genome tagging. Nature Methods. 2018;15: 598–600. doi:10.1038/s41592-018-0045-8

4. Soncini SR, Chandrashekarappa DG, Augustine DA, Callahan KP, O’Donnell AF, SchmidtI MC. Spontaneous mutations that confer resistance to 2-deoxyglucose act through Hxk2 and Snf1 pathways to regulate gene expression and HXT endocytosis. PLoS Genet. 2020;16: 1–30. doi:10.1371/journal.pgen.1008484

5. Schmidt GW, Welkenhuysen N, Ye T, Cvijovic M, Hohmann S. Mig1 localization exhibits biphasic behavior which is controlled by both metabolic and regulatory roles of the sugar kinases. Molecular Genetics and Genomics. 2020;295: 1489–1500. doi:10.1007/s00438-020-01715-4
