# Supplementary material for: Changing course: Glucose starvation drives nuclear accumulation of Hexokinase 2 in S. cerevisiae
Source: PLoS Genet. 2023 May 17;19(5):e1010745. doi: 10.1371/journal.pgen.1010745 (PMC10228819; doi:10.1371/journal.pgen.1010745)
Supplement: S2 Table — (DOCX) [file pgen.1010745.s014.docx]

**S2 Table.** Plasmids

| **Name** | **Description** | **Source** |
| --- | --- | --- |
| pRS315 | CEN *LEU2* | [1] |
| pRS315-Hxk2-GFP | Genomic clone of *HXK2* with 592 bp upstream of ATG and 373 bp downstream of the stop and a C-terminal fusion to EGFP; CEN *LEU2* | [2] |
| pRS315-Hxk1-GFP | Genomic clone of *HXK1* with 820 bp upstream of ATG and 704 bp downstream of the stop and a C-terminal fusion to EGFP. CEN *LEU2* | This study. |
| pRS315-Glk1-GFP | Genomic clone of *GLK1* with 939 bp upstream of ATG and 780 bp downstream of the stop and a C-terminal fusion to EGFP; CEN *LEU2* | This study. |
| pRS315-Hxk2^S15A^-GFP | The pRS315-Hxk2-GFP plasmid listed above had the S15A mutation introduced by site-directed mutagenesis with primers (Fwd: GAAAGGGTGCAATGGCCGATGTGCCAAAGG; Rev: TCGGCCATTGCACCCTTTCTGGCTTGTGGT). CEN *LEU2* | This study. |
| pRS315-Hxk2^S15D^-GFP | The pRS315-Hxk2-GFP plasmid listed above had the S15D mutation introduced by site-directed mutagenesis with primers (Fwd: GAAAGGGTGATATGGCCGATGTGCCAAAGG Rev: TCGGCCATATCACCCTTTCTGGCTTGTGGT). CEN *LEU2* | This study. |
| pRS315-Hxk2^K13A^-GFP | The pRS315-Hxk2-GFP plasmid listed above had the K13A mutation introduced by site-directed mutagenesis with primers (Fwd: TTAGGTCCAAAAAAACCACAAGCCAGAGCAGGTTCCATGGCCGAT; Rev: CAATTCCTTTGGCACATCGGCCATGGAACCTGCTCTGGCTTGTGG). CEN *LEU2* | This study. |
| pRS315-Hxk2^K13A,S15A^-GFP | The pRS315-Hxk2-S15A-GFP plasmid listed above had the K13A mutation introduced by site-directed mutagenesis with primers (Fwd: TTAGGTCCAAAAAAACCACAAGCCAGAGCAGGTGCAATGGCCGAT; Rev: CAATTCCTTTGGCACATCGGCCATTGCACCTGCTCTGGCTTGTGG). CEN *LEU2* | This study. |
| pRS315-Hxk2^K13A,S15D^-GFP | The pRS315-Hxk2-S15D-GFP plasmid listed above had the K13A mutation introduced by site-directed mutagenesis with primers (Fwd: TTAGGTCCAAAAAAACCACAAGCCAGAGCAGGTGACATGGCCGAT; Rev: CAATTCCTTTGGCACATCGGCCATGTCACCTGCTCTGGCTTGTGG). CEN *LEU2* | This study. |
| pRS315-Hxk2^Δ6-17^-GFP | We truncated pRS315-Hxk2-GFP residues 6-17 by PCR using primers (Fwd: TTTAGGTCCAGCCGATGTGCCAAAGGAA Rev: CACATCGGCTGGACCTAAATGAACCATTTTATTTAAT). CEN *LEU2* | This study. |
| pRS315-Hxk2-3V5 | Genomic clone of *HXK2* with 592 bp upstream of ATG and 373 bp downstream of the stop and a C-terminal fusion to 3V5; CEN *LEU2* | [3] |
| pRS315-Hxk2^S15A^-3V5 | The pRS315-Hxk2-3V5 plasmid listed above had the S15A mutation introduced by site-directed mutagenesis with primers (Fwd: GAAAGGGTGCAATGGCCGATGTGCCAAAGG; Rev: TCGGCCATTGCACCCTTTCTGGCTTGTGGT). CEN *LEU2* | This study. |
| pRS315-Hxk2^S15D^-3V5 | The pRS315-Hxk2-3V5 plasmid listed above had the S15D mutation introduced by site-directed mutagenesis with primers (Fwd: GAAAGGGTGATATGGCCGATGTGCCAAAGG Rev: TCGGCCATATCACCCTTTCTGGCTTGTGGT). CEN *LEU2* | This study. |
| pRS315-Glk1-3V5 | Genomic clone of *GLK1* with 939 bp upstream of ATG and 780 bp downstream of the stop and a C-terminal fusion to 3V5; CEN *LEU2* | This study. |
| pRS315-Hxk1-3V5 | Genomic clone of *HXK1* with 820 bp upstream of ATG and 704 bp downstream of the stop and a C-terminal fusion to 3V5; CEN *LEU2* | This study. |
| pRS315-Hxk2^Δ6-17^-3V5 | We truncated pRS315-Hxk2-3V5 residues 6-17 by PCR using primers (Fwd: TTTAGGTCCAGCCGATGTGCCAAAGGAA Rev: CACATCGGCTGGACCTAAATGAACCATTTTATTTAAT). CEN *LEU2* | This study. |
| pRS315-Hxk2^K7,8,13A^-GFP | The pRS315-Hxk2-GFP plasmid listed above had the K7A, K8A, and K13A mutations introduced by site-directed mutagenesis with primers (Fwd: AAATGGTTCATTTAGGTCCAGCAGCACCACAAGCCAGAGCGGGTTCCATG; Rev: CTTTGGCACATCGGCCATGGAACCCGCTCTGGCTTGTGGTGCTGCTGGAC). CEN *LEU2* | This study. |
| pSM3203-*TDH3pr*-Scs2-TM-mCherry | *TDH3* promoter- mCherry-Scs2-TM CEN *HIS3* AMP | [4] |
| pRS315-Hxk1^S15A^-GFP | The pRS315-Hxk1-GFP plasmid listed above had the S15A mutation introduced by site-directed mutagenesis with primers (Fwd: GGTCCAAAGAAACCACAGGCTAGAAAGGGTGCCATGGCTGATGTG; Rev: CAATTCCTTGGGCACATCAGCCATGGCACCCTTTCTAGCCTGTGG). CEN *LEU2* | This study. |
| pRS315-Hxk1^S15D^-GFP | The pRS315-Hxk1-GFP plasmid listed above had the S15D mutation introduced by site-directed mutagenesis with primers (Fwd: GGTCCAAAGAAACCACAGGCTAGAAAGGGTGACATGGCTGATGTG; Rev: CAATTCCTTGGGCACATCAGCCATGTCACCCTTTCTAGCCTGTGG). CEN *LEU2* | This study. |
| pRS315-Hxk1^K13A^-GFP | The pRS315-Hxk1-GFP plasmid listed above had the K13A mutation introduced by site-directed mutagenesis with primers (Fwd: TTAGGTCCAAAGAAACCACAGGCTAGAGCAGGTTCCATGGCTGAT; Rev: CAATTCCTTGGGCACATCAGCCATGGAACCTGCTCTAGCCTGTGG). CEN *LEU2* | This study. |
| pRS315-Hxk1^K7,K8,K13A^-GFP | The pRS315-Hxk1-GFP plasmid listed above had the K7A, K8A, and K13A mutations introduced by site-directed mutagenesis with primers (Fwd: AGATGGTTCATTTAGGTCCAGCGGCACCACAGGCTAGAGCGGGTTCCATG; Rev: CTTGGGCACATCAGCCATGGAACCCGCTCTAGCCTGTGGTGCCGCTGGAC). CEN *LEU2* | This study. |
| pRS315-Hxk1^D106A^-GFP | The pRS315-Hxk1-GFP plasmid listed above had the D106A mutation introduced by site-directed mutagenesis with primers (Fwd: CCATACCTTTGCCACCACTCAATCCAAGTAT; Rev: GATTGAGTGGTGGCAAAGGTATGGTTA). CEN *LEU2* | This study. |
| pRS315-Hxk1^Δ6-17^-GFP | We made an internal deletion of pRS315-Hxk1-GFP so that residues 6-17 were lost using PCR with the primers (Fwd: GTTCATTTAGGTCCAGCTGATGTGCCCAAGGAATTGATGGATG; Rev: CCTTGGGCACATCAGCTGGACCTAAATGAACCATCTTATTTTTTC). CEN *LEU2* | This study. |

**S2 Table References**

1. Sikorski RS, Hieter P. A system of shuttle vectors and yeast host strains designed for efficient manipulation of DNA in Saccharomyces cerevisiae. Genetics. 1989;122: 19–27. doi:10.1093/GENETICS/122.1.19

2. Hellemann E, Walker JL, Lesko MA, Chandrashekarappa DG, Schmidt MC, et al. Novel mutation in hexokinase 2 confers resistance to 2-deoxyglucose by altering protein dynamics. 2022. doi:10.1371/journal.pcbi.1009929

3. Soncini SR, Chandrashekarappa DG, Augustine DA, Callahan KP, O’Donnell AF, SchmidtI MC. Spontaneous mutations that confer resistance to 2-deoxyglucose act through Hxk2 and Snf1 pathways to regulate gene expression and HXT endocytosis. PLoS Genet. 2020;16: 1–30. doi:10.1371/journal.pgen.1008484

4. Zhou C, Slaughter BD, Unruh JR, Guo F, Yu Z, Mickey K, et al. Organelle-Based Aggregation and Retention of Damaged Proteins in Asymmetrically Dividing Cells. Cell. 2014;159: 530–542. doi:10.1016/J.CELL.2014.09.026
