# Supplementary material for: Changing course: Glucose starvation drives nuclear accumulation of Hexokinase 2 in S. cerevisiae
Source: PLoS Genet. 2023 May 17;19(5):e1010745. doi: 10.1371/journal.pgen.1010745 (PMC10228819; doi:10.1371/journal.pgen.1010745)
Supplement: Attachment — Submitted filename: Response to Reviewers_V2.pdf [file pgen.1010745.s018.pdf]

Response to Reviewers

Thank you to the Reviewers for their thoughtful and insightful comments. We feel that the revisions addressing these comments have improved the quality of the manuscript. We were pleased to hear that each Reviewer thought the work was convincing and experimentally sound. While we are actively pursuing studies to define the nuclear function of Hxk2, we cannot currently offer detailed mechanistic insight into this aspect of the story. We hope that the Reviewers appreciate that uncovering Hxk2's nuclear function is challenging and has largely eluded the field for >50 years. It will take at least two more years to address this question rigorously, which is why we feel it is beyond the scope of this initial submission.

*While the Reviewers felt the work did not meet the broad interest required for publication in PLOS Biology, given these comments, we feel the work in its current format would be a good fit for PLOS Genetics. The current paper serves the important function of correcting a long-standing controversy in the field, defining the environmental conditions and molecular players that allow Hxk2 to enter the nucleus, and demonstrating that the nuclear role of Hxk2 is not linked to the broad transcriptional changes associated with glucose repression of gene expression. Below we have a point-by-point response (italics, blue text) to each Reviewer's comments (*black text*).*

<u>Reviewer 1</u>

The O'Donnell team does a very thorough job of laying to rest an important controversy in the yeast glucose sensing field, i.e., the nuclear translocation of Hxk2 in response to external glucose cues. Their data robustly support the conclusion that Hxk2 is largely cytosolic in glucose-grown cells, and a portion of Hxk2 becomes nuclear localized upon glucose starvation. This simple observation is important as it contradicts previous work in the field. Plausible reasons for the discrepancy are given and, in some cases proven.

Thank you, Reviewer 1, for your kind words and deep appreciation for the study. We worked hard to ensure we did not miss some key facet of the past work that could help to explain the discrepancy between our studies and those done in the Moreno lab. Now that our current work defines the growth conditions and key features (i.e., Tda1 and residues in Hxk2) needed for Hxk2 nuclear translocation, we can focus future studies on Hxk2's nuclear function.

The authors subsequently revisit sugar kinase (hetero) dimerization and its relation to nuclear translocation. They find that Ser15 mutants do not affect nuclear translocation (again, in contrast to previous reports) but confirm that these mutations affect dimerization. They confirm and further previous observations that glucose binding leads to monomerization via expulsion of the N-terminal tail from the catalytic cleft of the adjacent kinases.

We, too, are excited to provide a plausible mechanism for why glucose drives the dimer-to-monomer transition in Hxk2 based on our MD simulations. To address comments made by Reviewers 2 and 3, we have moved much of this information into the supplemental data to streamline the narrative. However, we felt it was important to maintain this part of the work in the manuscript in some form to provide a needed context for glucose-induced dimer-to-monomer transition.

Their efforts then focus on the N-terminal 16 residues of Hxk2 showing that this region is required for nuclear exclusion (in glucose replete conditions). Finally, they focus on Lys13 and demonstrate that a K13A mutation leads to constitutive nuclear localization. Insinuated in the text is that K12 modification (dimethylation or sumoylation) could be responsible as analogous mutation in Hxk1 (in which K13 is apparently ubiquitylated) does not lead to nuclear localization, nor does mutation of D106, the residue that pairs with K13 to form a salt bridge.

The results conclude with data demonstrating that Hxk2 does not play a role in gene regulation, again in contrast to previously published works.

Thank you again. We felt it was particularly important to dispel the idea that Hxk2 functions in glucose repression of gene expression.

Overall, the paper is well written, albeit lengthy, and the robust yet largely contradictory data are important for the community and should be published.

Major Criticisms

i. My main concern with this work is the final model, which essentially lists the players involved but does not make any real attempt to explain how glucose mediates Hxk2 nuclear exclusion. 'This S15 phosphorylation is likely the predominant role that Tda1 plays in glucose-starved Hxk2 regulation, given that the S15D mutation restores Hxk2 nuclear accumulation in *tda1*∆ cells'. If this is true, why then does Hxk2-S15A relocalize similarly to WT Hxk2?

The Reviewer's excellent point prompted us to go back and assess the effect of the S15A mutant on Hxk2 nuclear localization in tda1∆ cells. As the Reviewer correctly indicated, the S15A mutation behaved the same way as the S15D allele and promoted Hxk2 nuclear accumulation in the tda1∆ background (see revised Fig 10A-C). We revised our statement to indicate that disruption of the Hxk2 dimer is likely the predominant role that Tda1 plays. However, since Tda1 phosphorylates S15 (Muller et al. 2022 Scientific Reports PMID 36302925) in both Hxk1 and Hxk2, this must be only one facet of regulation for Hxk2 nuclear translocation. Tda1's role in Hxk2's transition to the nucleus could be to lock Hxk2 in a monomeric state by phosphorylating S15, which primes it for a secondary step that helps Hxk2 move into the nucleus and/or accumulate there. Since the S15 mutants are still nuclear excluded in the tda1∆ background in high glucose medium, this regulation must have a second uncharacterized aspect.
Tda1 regulation must be distinct from the mechanism that keeps Hxk2 out of the nucleus in high glucose conditions, as monomeric mutants of Hxk2 do not readily enter the nucleus in glucose-replete conditions.

How does glucose maintain Hxk2 nuclear exclusion? It could be that: i) there is a nuclear export sequence that rapidly returns Hxk2 to the cytosol in glucose-grown cells, and this becomes occluded in low glucose, ii) there is a non-canonical NLS, and this is revealed in low glucose, or iii) there is a binding domain in Hxk2 that allows it to associate with a nuclear-localized protein and 'piggy back' into the nucleus in low glucose conditions. We have attempted to define a canonical NLS in Hxk2 (see S6A-D Fig), but this did not impact Hxk2 localization, and there are no other predicted NLSs in

Hxk2. The predicted NESs in Hxk2 (defined in Pelaez et al. 2009 JBC PMID 19525230 and Fernandez-Garcia et al. JBC 2012 PMID 23066030, both of which are from the Moreno lab) have been refuted in the literature (Fung et al., 2017 eLife PMID 28282025; Xu et al., 2012 MBoC PMID 22833565). In our hands, mutations in these sites generate unstable proteins that aggregate (data not shown). In the discussion, we now provide a more nuanced description of factors that could mediate nuclear exclusion in high glucose when describing the model figure.

'It is unclear if Snf1 is responsible for phosphorylating Tda1 to activate this kinase. However, it is tempting to speculate that this could be one mode of Snf1 regulation in this pathway.' Why, given that Snf1 played no obvious role in this regulation?

There are earlier reports in the literature that Snf1 regulates the Tda1 kinase in response to carbon-source switching (Oh S, et al. 2020 eLife PMID 33372657). Low glucose conditions activate the Snf1 kinase, and we find that Tda1 is hyperphosphorylated in response to glucose starvation (Fig 9E). In addition, several reports in the literature suggest Snf1 has a modest role in regulating the Hxk2 monomer-dimer balance, the most compelling of which is in Kaps et al. 2015 JBC (PMID: 25593311). In their Fig 2, they show that there is a shift from the monomer to the dimer form of Hxk2 when Snf1 is lost. This transition is even more striking in tda1Δ cells. Finally, there are new MS data sets using an AS-allele of Snf1 that monitors phosphorylation changes in response to glucose depletion. These data sets identify Hxk2 S15 as being Snf1 dependent (Caligaris, M. et al. 2023 eLife PMID 36749016), which may mean that there is some degree of Snf1 input on the S15 site via an indirect mechanism. The same data sets identify many Snf1-dependent phosphosites on Tda1. It could be that Snf1 activates Tda1 in a way that is redundant with other kinases, which would explain why Snf1 is not required in the same way that Tda1 is for Hxk2 nuclear transition. Together, these data suggest Snf1 could play a role in activating the Tda1 kinase that is redundant with other kinases or regulatory proteins. To clarify, we have modified our model figure (Fig 12) and have altered the text on lines 553-556 of page 26 and lines 737-742 of page 34 to better reflect this view.

'K13 is reportedly dimethylated or sumoylated [48,49], and either of these modifications could contribute to the regulated Hxk2 nuclear import.' Cannot this be assessed further e.g. by MS? At least assess the phenotype of an K13R mutation (which should retain the salt bridge but lose modification).

We thought the same thing and performed MS analyses of Hxk2 purified from yeast cells in high and low glucose conditions. However, we did not identify very many peptides that corresponded to the N-terminal domain of Hxk2, likely because this domain has many lysines and arginines, and so a trypsin digest would give rise to peptides that are too small to be captured by MS. We would need to redo this MS analyses using 'non-traditional' proteases. However, from this initial round of MS, we did observe dimethylation at K13 in the low glucose samples (3 peptides) and did not identify any dimethylation at K13 in the high glucose samples.

Irrespective of what MS results would show, we would need a secondary means to validate any identified posttranslational modifications. We tried to directly probe for dimethylation on Hxk2 using immunoblotting with an anti-methylation antibody (the

same one used to validate the MS identification of Hxk2 dimethylation in Zhang et al. 2016 PMID 27115613). We could not reproduce the result from this paper, likely due to a technical issue, as the anti-dimethylation antibody did not produce any specific signal in our hands.

Finally, we did as you suggested above and mutated the K13 to arginine. An arginine at this position will block sumoylation. However, arginine can be methylated, so it is somewhat unclear if this residue would still be methylated by the same enzyme. From these data, which we now show in S7D-F Fig, we find that the K13R mutation significantly increased nuclear Hxk2-GFP accumulation in high glucose conditions compared to WT Hxk2. However, there is not as much nuclear accumulation in high glucose for K13R as with K13A (S7D-F Fig). These results further support the idea that K13 is important; however, without a robust secondary validation of what modification occurs at this site (i.e., further MS analyses or a better methylation antibody), it remains unclear if modification of this residue is required. We have reworded our description of these putative modifications in the discussion to point out that the K13R mutation, which would prevent sumoylation, has lost glucose repression of Hxk2 nuclear translocation. If sumoylation is important, it could be required to exclude Hxk2 from the nucleus in high glucose conditions. Please see lines 749-768 on page 35 and the description of S7D-F in the results for the revisions to the manuscript.

ii. What is the physiological role of nuclear Hxk2? Answering this question would dramatically improve the impact of this manuscript. Are there phenotypes of tda1 cells that can be rescued by Hxk2-S15D for example?

Thank you for the thoughtful experimental suggestion. We will consider this in future studies that aim to define the nuclear function of Hxk2, which we, too, feel would be a valuable contribution to the field.

Reviewer 2

In the manuscript, Lesko et al. investigate a possible moonlighting function for the hexokinase Hxk2 in the nucleus of yeast cells. A substantial portion of the study is focused on presenting evidence counter to an existing manuscript in JBC by Moreno and colleagues on the role of Hxk2 in the nucleus in the glucose repression of gene expression.

There are indeed several aspects of this work that help correct the model for Hxk2 nuclear shuttling and function presented by the Moreno lab. We thank Reviewer 2 for bringing an important point to our attention with this comment. Because we did not sufficiently cite the works that our paper refutes, this Reviewer is left with the impression that we question only a single JBC paper. We have updated our citations throughout the paper to be more thorough. The Moreno lab model was established in over >12 publications spanning from 1998-2016 (~20 years as suggested by Reviewer 3 below) and has been cited 100s of times in the literature. To be candid, the effort it has taken our lab to test the many facets of the model is not insignificant and was not what we originally set out to do. However, this work has become a critical first step to help set the stage for our future endeavors that focus not only on the nuclear role of Hxk2 but also on the link between Hxk2 nuclear accumulation and resistance to a toxic analog of

glucose, 2-deoxyglucose. Correcting the existing model and providing new mechanistic insight is critical so we and others in the field can move forward.

Many of the methods and experiments employed are sound and clearly described. The authors have clarified that some Hxk2 appears to enter the nucleus, but in glucose starvation conditions as opposed to glucose replete conditions that was previously reported. They then identified several determinants of the nuclear localization/exclusion through mutagenesis experiments – they rule out a previous proposed role for S15 phosphorylation, and they report an involved kinase Tda1. Lastly, they show that there are minimal effects on the transcription of key genes when Hxk2 is deleted altogether.

Thank you for appreciating the new information on Hxk2 and its regulated translocation to the nucleus that this manuscript provides.

However, in the end, the authors have not elucidated the actual function of their observed nuclear pool of Hxk2 in glucose starvation conditions, which is a limitation of the current study.

As indicated above, we agree that this is an important element, but we cannot currently offer further insights. We are working to define the nuclear role of Hxk2; however, it will take considerably more experimental effort to thoroughly address this point, and we are not yet ready to provide mechanistic data supporting a specific model without further experimental refinement and validation. The publication of this work represents a key first step that will pave the way for other studies in the lab to define the impact of nuclear translocation on Hxk2 function and the role of nuclear Hxk2 in mediating resistance to 2-deoxyglucose, which is a toxic analog of glucose not discussed in this initial manuscript.

It is also not clear how well the extensive section on modeling and simulations fits the current manuscript – it reads as an analysis on the role of the N-terminal tail in dimerization, glucose binding, and catalytic activity and generates hypotheses that must be tested experimentally.

We appreciate this point, which was raised by Reviewer 3 as well. We, too, were somewhat concerned that this detailed section describing our MD simulations might detract from the flow of the manuscript. To help streamline the manuscript, we have moved the MD simulations data and description (now S4A-F Figs) into the supplement, where interested readers can peruse these models in conjunction with the size exclusion chromatography data.

Specific comments:

1. I am not sure the authors should describe their findings as a ‘paradigm shift’ (p. 3). To my knowledge, a role for nuclear localization of hexokinase was not well established or accepted to begin with. The manuscript reads like it is ‘clearing up’ a bunch of messy observations on a possible moonlighting function, unfortunately. A possible association of the enzyme with mitochondrial could be more physiologically relevant and interesting to study, as alluded to in the discussion. The authors mention that they did not observe

any mitochondrial Hxk2, but I would encourage them to examine this possibility more closely, or display some images with a mitochondrial marker as a figure or supplement.

We have considered that Hxk2 may co-localize with the mitochondria and used fluorescence microscopy to address this possibility. However, we did not detect obvious Hxk2-GFP co-localizing with the mitochondrial marker in either high or low glucose conditions (see Response to Reviewers Fig 1 below). In fact, we see some exclusion of the Hxk2-GFP signal from mitochondria in our imaging (denoted by white arrows). However, these data are not yet ready for publication, so we have not included them in this manuscript. Earlier studies in yeast suggest that Hxk2 is localized to the mitochondria, but these studies used biochemical fractionation and MS analyses, as in PMIDs 24769239, 31862471, and 16962558, and involved transitions to alternative carbon sources (i.e., glycerol) or specific stress inducers. It would be premature to consider this localization of Hxk2 in the current manuscript, but it is a facet of Hxk2 biology that should be further studied. Indeed, in conjunction with biochemical fractionation, a technique that could allow for selective detection of a mitochondrial pool, such as split GFP or BiFC, should be used so that the bright cytosolic fluorescence of Hxk2 would not interfere with the detection of a possibly smaller pool of Hxk2 localized to mitochondria.

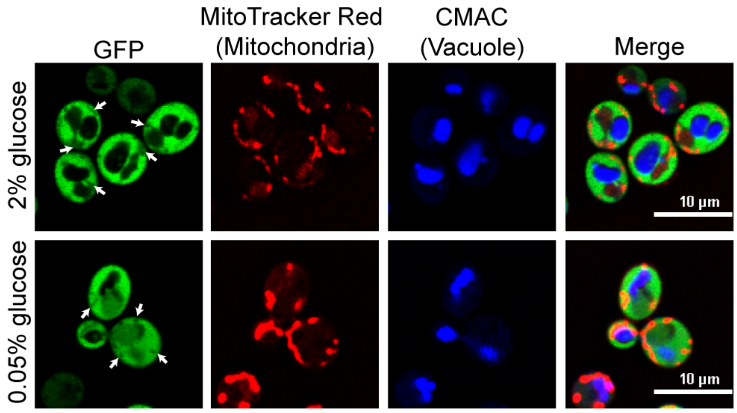

Reviewer Response Figure 1. Fluorescence microscopy of cells expressing a centromeric, plasmid-borne copy of Hxk2-GFP in *hxk2*Δ cells. Cells were grown in 2% glucose or shifted into 0.05% glucose for 2 hours. The vacuoles are marked in blue (CMAC), and the mitochondria are marked in red (MitoTracker Red staining). White arrows denote regions of dim/reduced Hxk2-GFP fluorescence that correspond to mitochondrial localizations based on the mitotracker staining.

2. The introduction seems focused on contradicting another group's work. Discussing and contradicting earlier reports is OK, but the majority of that should be deferred till the Discussion section perhaps. It is suggested that the authors should focus more on the outcomes, relevance and new knowledge contributed by their own study.

Our goal is to objectively present the commonly accepted model for Hxk2 nuclear regulation and compare it to our new model based on our latest findings. In our opinion,

the Introduction should inform the reader of what is already known in the field. In this case, we felt we had to summarize the Moreno model of Hxk2 nuclear-shuttling regulation. Unfortunately, the very real discrepancy between our model and the previous one needed to be addressed head-on and early in the paper to improve the chance that the future literature will be more accurate.

That said, we took the Reviewer's suggestion and either deleted or moved six sentences from the Introduction into the Discussion (sentences removed from paragraph spanning lines 75-84 on page 5). Hopefully, this strikes a better balance between directly addressing the issues while reserving some comparisons for the Discussion.

3. The authors have relied heavily on an approach that relies on the quantification of signal intensity of fluorescence-tagged proteins to determine the nucleus vs cytoplasmic abundance of proteins. They should validate that the addition of a large tag such as GFP is not affecting the localization or function of the protein of interest. They could consider complementing GFP localization studies with immunofluorescence analysis, and perform careful growth-rate measurements of GFP-tagged vs normal strains. In some of their Western blots it appears that there are some truncated forms of GFP-tagged proteins including Hxk2, possibly arising due to degradation, which could potentially impact the interpretation of some of their imaging experiments. Have they checked whether there might be possible compensation from Hxk1 or Glk1?

To address this concern, we performed growth curve analyses of cells lacking hexokinases (hxk1Δ hxk2Δ glk1Δ) and transformed with either vector, wild-type untagged Hxk2, or Hxk2-GFP. Note that the Hxk2 or Hxk2-GFP are expressed from CEN plasmids under the control of the Hxk2 promoter. Based on these assays, we see no difference in the growth rates of cells expressing Hxk2 or the GFP-tagged Hxk2 (S2A Fig), demonstrating that the GFP-tagged form is equally able to rescue growth on glucose when no other hexokinase is present. Thus, we conclude that the Hxk2-GFP is fully functional.

We performed immunofluorescence analyses of V5-tagged Hxk2. The results, while supportive of our model for Hxk2 nuclear regulation as defined using Hxk2-GFP, were somewhat inconclusive. In three replicate experiments using low glucose treated cells, very few cells remained with V5 signal after processing. We often saw a nuclear signal for DAPI and the Scarlet-tagged nuclear marker in the low glucose treated cells but almost no fluorescence for Hxk2-V5. We do qualitatively observe that Hxk2-V5 is nuclear excluded in high glucose and has increased nuclear fluorescence in low glucose (see Response to Reviewers Fig 2A). However, we have not included this figure in the manuscript as we do not feel it is robust enough in the absence of quantification. In addition, we subjected the Hxk2-GFP-containing cells to the immunofluorescence fixing and staining protocol. We found that Hxk2-GFP was nuclear excluded in high glucose and had distinct nuclear fluorescence in low glucose treatments; there were again many cells in the low glucose treatments that had weak or no Hxk2 fluorescent signal, which confounded quantification (Response to Reviewer's Fig 2B). It is unclear to us why this happened repeatedly in our low-glucose-treated cells during the IF protocol.

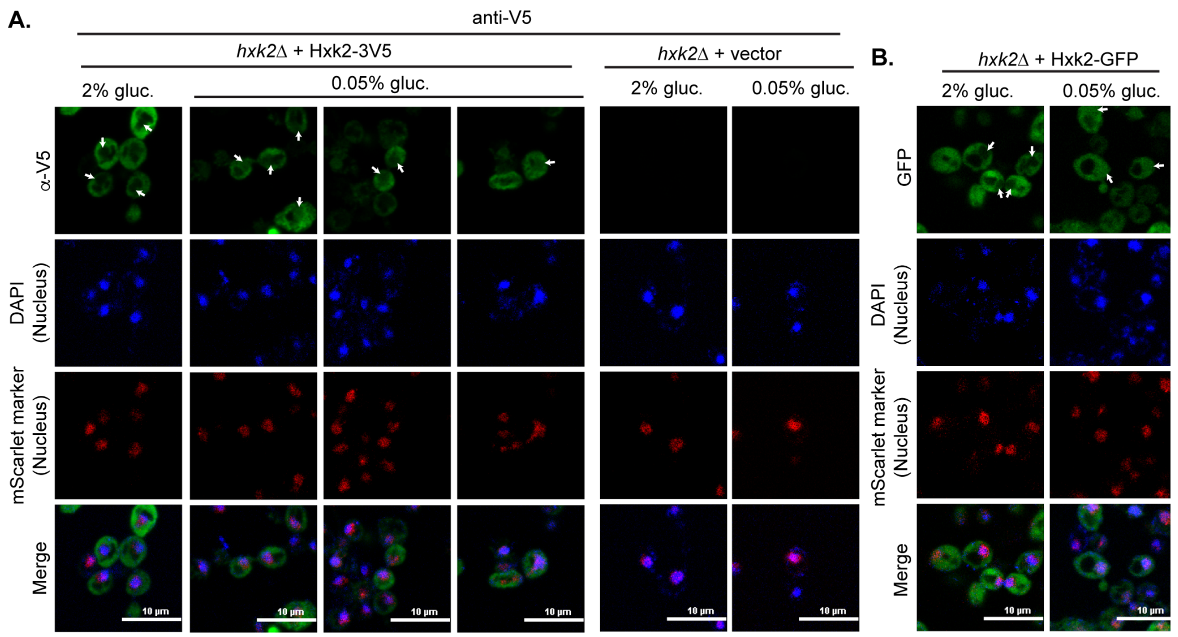

Response to Reviewer's Fig 2. Immunofluorescence of V5- and GFP-tagged Hxk2. A) Hxk2-V5 or B) Hxk2-GFP immunofluorescence of cells grown in high glucose or shifted into low glucose for 2 h. DAPI staining and the mScarlet marker denote the nucleus. White arrows indicate regions of nuclear exclusion or staining in each column. Note the presence of many cells with nuclear staining for DAPI and the Scarlet marker but lacking discernible V5 or GFP fluorescence.

Finally, we included the immunoblot in S2E Fig to help demonstrate that the alterations in nuclear Hxk2-GFP signal at 2h post low-glucose shift (as observed in many experiments throughout the manuscript) was not simply due to increased cleavage of GFP from Hxk2-GFP. In fact, there is no increase in the free GFP breakdown product until cells have had prolonged incubations in glucose starvation conditions (~8-24 h; S2E Fig). Further, based on the Western blots (shown in S2E Fig and the newly added immunoblot in S7G Fig), there is very little GFP breakdown product (<5%) compared to the abundance of the intact Hxk2-GFP. Importantly, the amount of free GFP observed in these immunoblots does not increase when cells are shifted into low glucose conditions, nor is it elevated with the Hxk2^{K13A} mutant, which has increased nuclear fluorescence in high glucose relative to the WT Hxk2-GFP. From this, we conclude that the increased nuclear fluorescence for Hxk2 in low glucose or in the K13A mutant is not due to increased free GFP in the cells. We added text to clarify this point at lines 242-244 on page 12 and 470-472 on page 22.

4. A substantial portion of the results is homology modeling and molecular dynamics simulations to examine mechanisms of dimerization and the role of the N-terminal tail. It is a bit unclear how this section addresses a possible role of Hxk2 nuclear localization, and does not fit well with the rest of the study. Moreover, the manuscript would benefit from a more detailed comparison of Hxk1 and 2, both from a discussion and experiment point of view. The authors briefly mention that Hxk1 and 2 are closely related paralogs with 89% similarity in their amino acid sequence. But then, why Hxk1 doesn't localize to the nucleus? The N-terminal tail clearly does not explain it. Is it appropriate to use the structure of K. lactis Hxk1 then for such modeling studies?

We have moved the molecular dynamics simulation data into the supplemental (S5 Fig) to help streamline the text while maintaining the idea that glucose may drive the dimer-to-monomer transition for Hxk2 by disrupting key N-terminal associations with the opposing monomer.

We have provided a more detailed comparison of Hxk1 and Hxk2, as suggested. We added a new S4 Fig that shows a full sequence alignment (S4A Fig) for ScHxk1, ScHxk2, and KlHxk1, as well as figures demonstrating how these structures compare to one another (S4B-D). We added information on the comparisons between Hxk1 and Hxk2 to the discussion (see lines 749-768 on page 35). Despite their high degree of sequence conservation/similarity, there are structural differences between Hxk1 and Hxk2 (see S4B Fig). In addition, there are posttranslational modifications that differ between Hxk1 and Hxk2 that could impact nuclear translocation. Some of these posttranslational modifications occur within the N-terminus of Hxk2. Since we find that mutation of Hxk2 N-terminal residues can promote Hxk2 nuclear partitioning, we posit that modifications at these sites may be important regulators. At this time, we do not know why Hxk2 localizes to the nucleus while Hxk1 does not, but it could be because Hxk1: i) lacks an NLS that is present in Hxk2, ii) contains a robust nuclear export sequence, iii) binds some element that prevents it from accumulating in the nucleus or iv) fails to bind to the element that may help Hxk2 translocate into the nucleus.

Finally, there was a genome duplication event in Saccharomyces cerevisiae, so it often has two paralogous copies of genes that have only a single representative in Kluyveromyces lactis, which did not undergo the genome duplication. ScHxk1 and Sc Hxk2 are paralogs that arose from this genome duplication in Saccharomyces cerevisiae, and KlHxk1 is the only ortholog of both genes in this species. As seen in the structural comparison (S4B Fig), KlHxk1 is an excellent structural model for ScHxk2. While the naming may be misleading, KlHxk1 is more similar to ScHxk2 than it is to ScHxk1 (S4A Fig). Structurally, the monomer of KlHxk1 overlays nearly perfectly on the ScHxk2 structure (S4B Fig). However, there are differences between the KlHxk1 and ScHxk1 sequences, most notably in an alpha helix that runs along the top of the structure (S4B and D Fig). We have adjusted the wording in the text associated with this new supplemental figure (see lines 297-314 on page 15) to better convey why KlHxk1 is the best choice when modeling ScHxk2.

5. Why did the authors opt to use ion exchange chromatography instead of size exclusion for determining the oligomeric states of Hxk2? Is there a possibility that Hxk2 could dimerize with Hxk1 or Glk1?

This is a typographical error on our part. We correctly indicated in the Methods section that we used size exclusion chromatography but somehow indicated that it was ion exchange chromatography in the results section. We have corrected this language throughout the text. We apologize for the confusion, and thanks for catching this mistake.

As for the question of Hxk2 dimerization with Hxk1 and Glk1, we show in S1G Fig that differentially tagged Hxk1 and Hxk2 can copurify with one another, albeit to a lesser extent than what we observed when we used two differentially tagged forms of Hxk2.

We did not explore the possibility of Hxk2 and Glk1 forming multimers. This is an interesting idea that we may consider in the future.

6. They suggest that three lysines among residues 7-16 could be critical for an NLS. However, mutation results in constitutive nuclear localization as opposed to exclusion, so it is difficult to follow their logic here. I would caution against the phase 'constitutive nuclear localization' as it implies the entire pool of Hxk2 is nuclear, but I believe the authors are referring to the constitutive localization of a small pool of the enzyme that is not responsive to glucose.

*Now we see why there could have been confusion in reading this section. To be clear, we are **not** proposing that amino acids 7-16 constitute an NLS. The deletion mutation of these amino acids was first generated and used by the Moreno group, who defined this region as an NLS (see Herrero et al. 1998 PMID 9738454). This mutation is referred to as both Hxk2ΔK^{7}M^{16} and Hxk2^{WRF} by the Moreno group in distinct publications. It was reported to i) prevent Hxk2 nuclear accumulation, ii) impair glucose-repression of SUC2 gene expression, and iii) retain full Hxk2 catalytic function. We were, therefore, surprised to find that Hxk2$^{\Delta 7\text{-}16}$ is not only present in the nucleus but has a nuclear signal when cells are grown in either high or low glucose conditions. Our findings refute the earlier findings for this mutant and dispel the idea that this region contains an NLS. We have reworded lines 393-401 on page 19 to reflect these changes more accurately. Hopefully, this rewording clarifies the differences between data from the past work and our current study.*

As stated below in response to another of Reviewer 2's comments, we used many computational approaches to try and identify a putative NLS for Hxk2. Using site-directed mutagenesis and fluorescence microscopy, we assessed a second putative K/R-rich NLS in Hxk2 (see S6D-G Fig). However, this mutant maintained the same nuclear exclusion and accumulation pattern in high and low glucose, respectively, as observed for WT Hxk2.

Regarding the term 'constitutive nuclear localization,' we have removed this language throughout the Results text and figure legends per the Reviewer's suggestions.

7. What are the kinetics of the cytosolic Hxk2 recovery in FRAP experiments?

We did not set a region of interest to bleach within the cytosolic pool of Hxk2 in our FRAP experiments, so we do not know the answer to this question. Unlike with nuclear bleaching, we anticipate that the pool of cytosolic Hxk2 is freely diffusing. If a region of interest in the cytosol were bleached, we would predict that the signal recovery would be limited only by the diffusion rate, returning rapidly to maximal signal intensity.

8. The authors could consider fusing a strong nuclear export sequence (NES) to Hxk2 and carefully assess whether there is any consequence. Alternatively, they could identify a mutant (in a bona fide NLS?) that completely excludes Hxk2 from the nucleus in glucose starvation conditions, and then determine the consequences. These would be more definitive experiments to address what role, if any, does this nuclear pool of Hxk2 serve under glucose starvation?

We thank the Reviewer for this suggestion. Experiments are ongoing in our labs to assess the functional impact of fusing a strong NES and strong NLS to Hxk2. However, we cannot offer functional insight into the role of the nuclear pool at this time.

However, we tried to define a bona fide NLS in Hxk2 (as opposed to the previously reported NLS in the Hxk2 N-terminus, which we have debunked herein) using NLS prediction software. We found that mutation of this second predicted NLS did not alter the distribution of Hxk2 in cells (S6A-D Fig). There are no other predicted NLS in Hxk2 based on any software we have used. The localization of Hxk2 to the nucleus could be regulated by a bi-partite or non-canonical NLS, which are very difficult to define. Alternatively, localization could be controlled by a 'piggy-backing' mechanism, whereby Hxk2 binds to a second protein that itself contains an NLS. This piggy-backing model is how human GCK is thought to arrive in the nucleus (see discussion lines 770-779), and currently, this is the model we favor for Hxk2 in yeast. We are currently using MS analyses of factors copurifying with Hxk2 in response to low glucose treatment to help define possible candidates for this translocation mechanism.

Reviewer 3

"Glucose repression" of gene expression is an important and well-studied regulatory phenomenon in yeast. The main mechanism can be boiled down to glucose regulation of the AMP-activated protein kinase Snf1, which regulates activity of the Mig1 repressor that represses transcription of many (most?) glucose-repressed genes. Several other proteins play various roles in glucose repression, among which is a hexokinase encoded by Hxk2.

One obvious idea for the role of Hxk2 in glucose repression is that it is involved in the generation of a signal through its role in glucose metabolism. Over the years there were hints that Hxk2 has a regulatory function independent of its catalytic activity. The initial evidence for this (from Entian and from Thevelien) was merely suggestive, but the Moreno lab built a case that HXk2 is part of the repressor bound at promoters of glucose repressed genes. I was always skeptical of their model, mostly because Hong Ma and David Botstein found that the extent of glucose repression in various hxk2 mutants is (invariably) correlated with the enzymatic activity of their Hxk2 proteins (MCB 9:5643-9, PMID: 2685572). The simplest interpterion of that result is that Hxk2 influences glucose repression through its generation of glucose-6-P, which presumably affects the signal received by the Snf1 protein kinase. I always wondered what to make of the evidence from the Moreno lab (presented in several papers over almost 20 years) showing that Hxk2 is in the nucleus, binds to the Mig1 repressor, and is present at promoters of glucose-repressed (Mig1-boung) genes. This paper submitted for publication in PLOS Biology presents convincing evidence that refutes nearly all aspects of the model built by Moreno et al.. The main conclusions are:

1. Hxk2 is not in the nucleus when glucose is abundant, contradicting results from the Moreno lab. In fact, it seems excluded from the nucleus when glucose is abundant, because glucose starvation pushes it into the nucleus (Figs. 1,2), exactly the opposite of what Moreno et al. reported.
2. The N-terminus of HXk2 contains a sequence (residues 7-16) that excludes it from the nucleus when glucose is abundant (Figs. 5,6), opposite from what Moreno's lab reported.

3. Substitutions of S15 do not affect the nuclear location of Hxk2, contradicting results from the Moreno lab and suggesting that phosphorylation of S15 plays no role in the nuclear localization of HXk2 (Fig. 3, supported by results of experiments presented in Fig. 6C-E).
4. Pseudo-phosphorylation of S15 (S15>D) prevents Hxk2 dimer formation, suggesting that S15 phosphorylation regulates the monomer-dimer state of Hxk2 (Fig. 3F)
5. Glucose binding to Hxk2 promotes monomer formation (Fig 4).
6. Monomerization of HXk2 does not drive its nuclear localization (Figs. 6, 7, supported by various other experiments), in contrast to Moreno's model.
7. Neither Mig1 nor Snf1 are required for Hxk2 nuclear localization (in low glucose) (Fig. 8), in contrast to what the Moreno lab reported.
8. The Tda1 protein kinase is required for Hxk2 nuclear localization (in low glucose) (Fig. 8). But it is not required for nuclear localization of Hxk2 S15>D (Fig 10), suggesting that phosphorylation of S15 contributes to nuclear localization.
9. Hxk2 is involved in regulating the expression of very few genes (Fig. 11).

The evidence for all of that seems unimpeachable to me; I really found nothing in it to question. The authors come up with some plausible explanations for why their results are different from those of the Moreno lab. My guess is that it's simply because their imaging is much better than Moreno's, the methos having advanced so much since Moreno et al. did their imaging.

Thank you, Reviewer 3, for your kind words. We are happy to hear that you support the conclusions of this work. We do think the improved imaging could explain why we came to very different conclusions than the Moreno lab. It is also likely that the processing used by the Moreno lab to allow more robust DAPI staining created some issues with data interpretation. As described below in the response letter, S1F Fig is our attempt to directly reproduce the findings from these earlier publications.

Where does this leave us regarding the role of Hxk2 in glucose repression? If Hxk2 is not in the nucleus when glucose is abundant – and I'm convinced it's not based on the authors' results – then Moreno's model that Hxk2 directly collaborates with Mig1 to repress transcription cannot be correct (though I don't know how to account for Moreno's ChIP evidence that Hxk2 is present at glucose repressed genes). Unfortunately, the authors shed no light on what Hxk2 is doing to effect glucose repression, though that is certainly beyond the scope of this paper. I submit that refuting the Moreno model is a significant contribution to the literature. But this paper brings us no closer to understanding how Hxk2 is involved in glucose repression.

We agree with these points raised by Reviewer 3. We are pleased to hear that changing the existing model for Hxk2 nuclear portioning is a significant contribution to the field in the Reviewer's estimation. We also agree that defining the nuclear role of Hxk2 is critical to the field. Our future work will address this role, but as stated earlier, we cannot offer a definitive function at this time.

In fact, the authors present evidence that suggests that Hxk2 does not 'play a role in regulating glucose repression (line 671 of the ms.). That confuses me. Mutants of hxk2 were among the earlier glucose repression resistant mutants that were obtained, and

HXK2 clearly plays a role in glucose regulation of several well-studied genes (HXT1 and HXT4: Table 5 of PMID: 7862149; SUC2 and CYC1: PMID: 3540605; are just a few of the many examples in the literature). After seeing very few genes change expression when Hxk2 is deleted (Fig. 11), and seeing none of the genes whose expression has been shown to be affected by Hxk2 in the list of most highly glucose-repressed genes (Fig. 11F), I'm, well, confused. It seems to me we're back to square one on the issue of Hxk2's involvement (if any) in glucose repression. (But at least we're no longer going down a wrong path.)

We see now that we did not properly describe our findings here. We have revised lines 615-618 on page 29 and added S11A-B to improve clarity. We appreciate that the changes in expression of Hxk1, Hxt1, Hxt2, Hxt3, Hxt4, Suc2, and Cyc1 in hxk2∆ cells were reported broadly in the literature. To be clear, we do find modest changes in expression between WT and hxk2∆ cells on high glucose for this handful of genes (S11A), which is consistent with the publications cited above by Reviewer 3. We did mention this in the Results section of the initial draft of the paper, but now we have expanded on this concept.

However, the modest expression changes of these few genes in hxk2∆ cells relative to WT are outliers in the RNAseq data set, and they do not change in expression as much as they do when glucose repression is truly lost (i.e., when cells are shifted to low glucose). As suggested by this Reviewer, it is true that these six genes are NOT the genes whose expression changes the most in response to low glucose.

To clarify, we identified the top Mig1 binding genes from Rossi et al. 2021 Nature (PMID:33692541) and filtered these for genes whose expression change was >2-fold in response to low glucose treatment. We then compared the expression profiles for these genes across conditions (high or low glucose in WT or hxk2∆ cells) and found no difference in the transcript profiles (S11B Fig).

My only significant criticism of this paper is that it seemed a bit disjointed. There are two main stories: Hxk2 subcellular localization and Hxk2 structure. The latter is related to the former because the Hxk2 monomer-dimer state was linked to its nuclear localization, but the authors could have delinked those issues (as they conclusively do) more concisely. The structural modeling and molecular dynamic simulations seem to me to be a distraction, more appropriate for a paper for a more specialized audience.

We appreciate the Reviewer's points. Based on these comments and those of Reviewer 2, we have moved the MD simulations and their detailed descriptions into the supplement (please see the response to Reviewer 2 above for a more detailed explanation). However, we retain this information in the supplement because we believe it provides a plausible explanation for why glucose binding to Hxk2 stimulates the transition to the monomer. Further, the role of the N-terminal region that the simulations suggest is in complete disagreement with its function as a putative NLS.

We retained the Hxk2 dimer model as it provides a critical structural framework for understanding the impact of the N-terminal and dimer-breaking mutants used in this study. We discuss more aspects of the model and its relevance to Hxk2 in our response to Reviewer 2 above.

I'm somewhat surprised this paper was submitted for publication in PLOS Biology, which I believe is intended for a relatively broad audience. The paper will be of high interest to a fairly limited audience interested in glucose repression in yeast. The authors make a valiant effort to make the story more broadly relevant by citing the mammalian work on this subject. Indeed, people interested in hexose kinases and glucose sensing/metabolism in other organisms, including humans, will likely appreciate that the situation has been clarified by this work (i.e., that yeast is less an outlier than the Moreno model made it seem). Whether that is sufficient to justify publication in a general-interest journal is, of course, the editor's decision.

We have opted to shift the submission to PLOS Genetics and feel that this journal's readership will appreciate the work's scope. We think researchers interested in glucose metabolism, spanning from yeast to man, will find the work useful.

A few minor comments/questions/suggestions:

Lines 704-5: 'The ~150 million years of evolution that separates yeasts and humans'? Isn't it more like 1000 million years (give or take a few hundred million)?

You are right. We changed it to ~ 1 billion to make things easier and added the following citations (PMIDs: 25999509 and 1549441).

Lines 727-9: 'We find that Hxk2 shifts to a monomer when glucose binds, confirming earlier studies that demonstrate a dramatic increase in the association constant of Hxk2 dimers when glucose is present [33]'
Shouldn't it be 'a dramatic decrease in the association constant'?

It most definitely should be a decrease. We made this change. Many thanks for catching the mistake!

Lines 197-9: Suggest changing to: Therefore, if there was a significant accumulation of Hxk1 in the nucleus we expected we would have been able to detect it in the glucose starvation conditions, and we did not (Fig 1A-C).

Thank you for the added clarity; we have revised these lines as suggested.

Fig. 1E: The cells were grown in high glucose?

For the co-purification studies, each of the hexokinases was expressed from its own promoter, and the cells were grown in a high (2%) glucose-containing medium. For clarity, we added that information to the figure legend and the methods (pg 37; lines 845-847). We did the co-purifications under low glucose and in response to 2-deoxyglucose (see Response to Reviewer's Fig 3 below), which are included for your consideration. We do not think there is any difference in the amount of Hxk2 co-purification in response to these changing conditions.

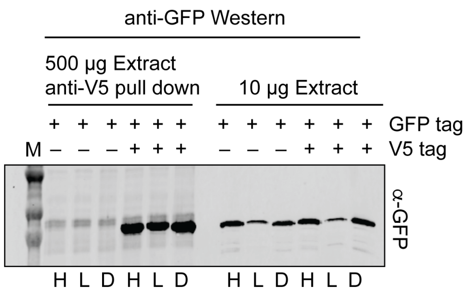

Reviewer Response Figure 3. Hxk2 copurifies with itself in a range of conditions. Cells containing the indicated tagged forms of Hxk2 were grown in either high glucose (H), shifted to low glucose for 2 h (L) or had 0.2% 2-deoxyglucose added for 2 h (D) and protein extracts were made. The V5-tagged form was immunoprecipitated from these cells and co-purification of the GFP-tagged form assessed. These data support a model where Hxk2 can multimerize in high glucose, low glucose or the presence of 2DG.

S1F Fig: I can't make any sense of this.

*The lack of clear interpretation of this data is sort of the point of this figure. We think that the way the Moreno lab was treating the cells in glycerol made it impossible to identify localization changes, as the glycerol incubation muddied the Hxk2 partitioning. What we have done in S1F Fig is comparable to Figure 1A in PMID: 18588509, or Figure 1A from PMID: 19525230 from the Moreno lab. Screen captures of these figures are included below to make comparisons easier. We *think* we can still see a bit of nuclear exclusion in cells grown in 2% glucose and shifted into glycerol before imaging (S1F Fig), but it is hard to discern and impossible to be confident in a qualitative assessment of these data. The 0.05% glucose-glycerol-shifted cells do seem to have a decent nuclear signal overall (suggesting nuclear accumulation of Hxk2), but again it is just not as clear as the live cell imaging. Even the vacuole exclusion of the Hxk2-GFP signal, which we routinely see in live cell imaging, is difficult to identify when cells are processed as in S1F Fig.*

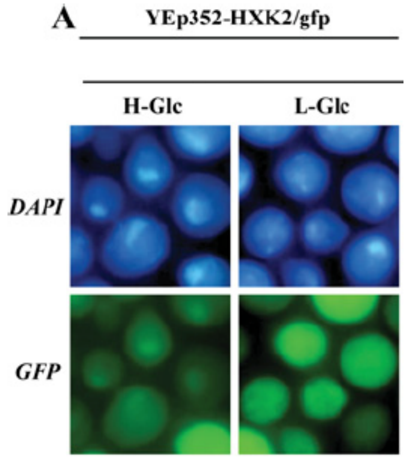

Taken from Figure 1A of Riera et al. (PMID 18588509) where cells were imaged as in S1F Fig of Lesko et al. (under review). Cells were grown in high glucose (H-Glc) or shifted to low glucose (L-Glc) but prior to imaging were suspended in 80% glycerol containing DAPI.

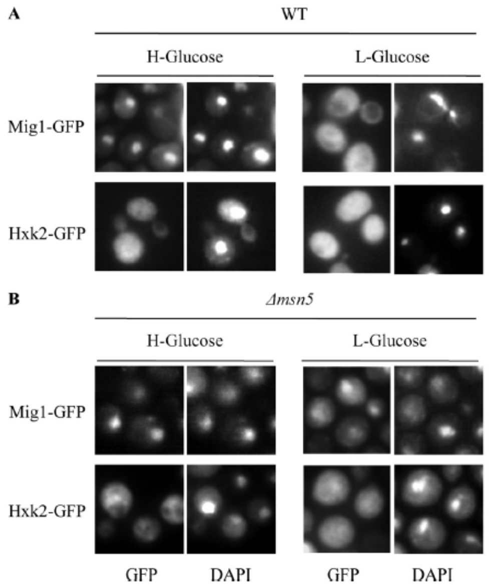

Taken from Figure 1A-B of Pelaez et al. (PMID: 19525230) where cells were imaged as in S1F Fig of Lesko et al. (under review). In panel 1A, distinct localization of Hxk2 to the nucleus in either high or low glucose conditions is difficult to discern.

Reviewer: Mark Johnston
