# Supplementary material for: Changing course: Glucose starvation drives nuclear accumulation of Hexokinase 2 in S. cerevisiae
Source: PLoS Genet. 2023 May 17;19(5):e1010745. doi: 10.1371/journal.pgen.1010745 (PMC10228819; doi:10.1371/journal.pgen.1010745)
Supplement: Attachment — Submitted filename: Response to Reviewers_PG.pdf [file pgen.1010745.s021.pdf]

*Thank you again to the Reviewer for their thoughtful comments. Below we have a point-by-point response (italics, blue text) to each Reviewer's comments (*black text*).*

Review of Lesko et al.

The authors have adequately addressed my comments (and, it seems to me, those of the other reviewers).

I'm still mystified that the authors find that *HXK2* seems to play no role in glucose repression, even though numerous studies of many individual glucose repressed genes over many (50?) years suggest that it plays a significant role in this process. Nevertheless, I believe this paper will be a worthy contribution to the literature.

We appreciate that this is a big change in the view for Hxk2 function. However, as you previously pointed out, the earlier data from the Botstein lab supported the idea that the modest changes in transcription that were observed in hexokinase 2 mutants correlated well with enzyme activity and therefore likely represented transcriptional alterations linked to loss of Hxk2 enzymatic function rather than a 'moonlighting' function as a transcriptional repressor. Unlike in other systems, there appear to be no 'separation of function' alleles, where Hxk2 enzymatic function is intact and only the transcriptional changes linked to Hxk2 are observed. The one mutation, 'WRF' that appeared to separate catalytic function from gene expression changes (based on data from the Moreno lab), does not exhibit any of these phenotypes when we reassessed it.

In addition, all past studies focused on a few genes as readouts of glucose derepression. To our knowledge, none compared transcriptional changes on a global level in response to hxk2 mutations and a switch to glucose abundance. As we point out in the manuscript, there are some modest changes in gene expression in hxk2Δ cells and those that have been observed in the literature are supported here in many cases, including the gene expression changes reported for Suc2, Hxk1, and Hxt1. However, these changes are very small in magnitude compared to the transcriptional changes found in cells that have truly lost glucose repression (i.e. in response to glucose starvation). When the transcriptional data are compared on a whole-genome scale, the minor changes in gene expression identified in hxk2Δ in the past are not reflective of the transcriptional changes observed during a global loss of glucose repression. There is no evidence for Hxk2 at these chromosomal locations in any of the ChIP-seq datasets either.

While there are some modest transcriptional changes in hxk2Δ cells based on our studies and those of others, these changes do not reflect a global loss of glucose repression in the absence of this enzyme and do not appear to be linked to the nuclear propensity of Hxk2. Unlike Mig1, which clearly has a robust role in glucose repression, Hxk2 is not operating as a cofactor for this transcription factor in regulating glucose repression.

A few comments and suggestions the authors may want to address:

Two things confused me. First:

> A second layer of glucose-induced regulation must exist for Hxk2 because Hxk2$_{S15D}$ and Hxk2$_{S15A}$ are nuclear excluded **even in glucose-grown cells**.

"Even in glucose-grown cells" doesn't make sense to me because Hxk2 is normally nuclear excluded in glucose-grown cells.

We have altered the language here to clarify (see lines 673-680). The point we are trying to make is that in low glucose, phosphorylation of Hxk2 at S15 is occurring via the Tda1 kinase and this is needed to split Hxk2 into its monomeric form. However, that is not enough to drive Hxk2 into the nucleus in high glucose conditions, as we see no change in the distribution in glucose replete conditions for Hxk2-S15D and S15A. Therefore, in glucose-grown cells there must be some other additional regulatory feature that keeps Hxk2 out of the nucleus. This regulation is disrupted in the Hxk2-K13A or K13R mutations.

Second, In two places in the ms. I'm told that Hxk2 forms dimers (and monomers) in high glucose, but forms predominantly monomers in low glucose:

> Rigorous biochemical analyses demonstrate that in **glucose-replete conditions**, a balance of **monomeric and dimeric** Hxk2 exists, but Hxk2 shifts to **predominantly monomeric when glucose is restricted** [29–32].

> In a **glucose-rich** medium, Hxk2 exists in a balance between **dimeric and monomeric** species [32,33]. Upon **glucose starvation**, this balance shifts toward the **monomeric** state [32,33].

But then I'm told that high glucose "destabilizes the dimer" and that "low glucose encourages dimerization".

> In **high glucose concentrations**, bound glucose might disrupt N-terminal-tail binding within the catalytic pocket to **destabilize the dimer** (Fig 4A). Alternatively, N-terminal-tail binding may prevent glucose binding in **low glucose concentrations, encouraging dimerization.**

This is really a terrific point, and we thank the Reviewer for raising it. We need to clarify the monomer-dimer regulation as it exists in vitro vs in vivo.

<u>In vitro</u>: Purified Hxk2 analyzed in the absence of glucose is a dimer. Addition of glucose impedes dimer formation, driving Hxk2 to its monomeric state. We find this to be the case with our size exclusion chromatography (Figure 4) and the Kriegel lab has reported this same finding, adding in the details for Ka (as described on lines 407-413 and references therein).

<u>In vivo:</u> In Kaps et al (PMID: 25593311; Figure 5) they show that:

1) *In 2% glucose, Hxk2 exists as a balance between monomer and dimer species.*
2) *When you shift cells from 2% glucose into 0.1% glucose or a glucose/glycerol combo, the equilibrium between the Hxk2 monomer-dimer shifts towards the monomer.*
3) *This monomer-dimer balance shifts dramatically towards the dimer in tda1Δ (aka ymr291wΔ) cells and is maintained as a dimer, even in low glucose conditions.*

From these data, we can conclude that in vivo, when glucose is present there is a monomer-dimer transition that is happening to keep this balance. However, when you shift cells into low glucose conditions, the Tda1 kinase can phosphorylate Hxk2 to prevent its dimerization. In vivo, it is this layer of Tda1-directed, post-translational regulation that keeps Hxk2 in a monomeric state. In the absence of that posttranslational regulation, as we would expect based on the in vitro data, Hxk2 is dimeric in glucose restricted cells as demonstrated by the Kriegel lab.

Based on the Reviewer's suggestion, we modified lines 78-81, 187-195, 308-319 and 673-680. We hope this helps clear up any confusion on these points.

I like to offer authors suggestions for captivating titles (see https://genestogenomes.org/how-to-write-titles-that-tempt/). Here's my suggestions for this paper:

> Whither Hexokinase 2? To the nucleus when glucose is scarce, but not to regulate gene expression.
>
> Glucose regulation of gene expression in S. cerevisiae: no role for Hexokinase 2.
>
> No role in glucose regulation of gene expression for Hexokinase 2 of *S. cerevisiae*

Thanks for pointing out this paper. It was an interesting read, and we will keep it in mind when crafting future titles. While we think the title 'Whither Hexokinase 2? To the nucleus when glucose is scarce, but not to regulate gene expression' would be an EXCELLENT title for a Commentary on this work, we will pass on that suggestion for the title of the primary paper.

In considering the 'rules' suggested by this article, we have modified the title to:

Changing course: Glucose starvation drives nuclear accumulation of hexokinase 2 in S. cerevisiae

We hope you find this more compelling.

Finally, the ms. is long. I suspect it could be modestly shortened (and made clearer) with aggressive editing. While editing the ms., I urge the authors to follow Strunk and White's dictum: "avoid needless words." Below are my suggestions of text that can be deleted. (I realize this will achieve only modest shortening, but I thought I should provide some examples. And these are only suggestions; I hope they're useful.)

Thanks for the suggestions. The paper was a bit wordy (as are most things I write). In opposition to Strunk and White's hatred of the adverb, do like them and enjoy reading them in other

people's papers too. However, since they are relegated as 'needless' by S&W, we have removed them and worked on tightening up the language throughout the document. See the tracked changes in one of the uploaded versions of the manuscript for details on these changes. We reduced the word count from 11,724 to 10,720 (a reduction of ~10%) and incorporated nearly all of the suggestions outlined by this Reviewer. We even got rid of the supplemental data on Reg1 as suggested. ***Thank you for your help in making the manuscript clearer!***
