# Supplementary material for: Changing course: Glucose starvation drives nuclear accumulation of Hexokinase 2 in S. cerevisiae
Source: PLoS Genet. 2023 May 17;19(5):e1010745. doi: 10.1371/journal.pgen.1010745 (PMC10228819; doi:10.1371/journal.pgen.1010745)
Supplement: Attachment — Submitted filename: Review of Lesko et al_final.docx [file pgen.1010745.s019.docx]

Review of Lesko et al.

The authors have adequately addressed my comments (and, it seems to me, those of the other reviewers).

I’m still mystified that the authors find that *HXK2* seems to play no role in glucose repression, even though numerous studies of many individual glucose repressed genes over many (50?) years suggest that it plays a significant role in this process. Nevertheless, I believe this paper will be a worthy contribution to the literature.

A few comments and suggestions the authors may want to address:

Two things confused me. First:

A second layer of glucose-induced regulation must exist for Hxk2 because

739 Hxk2S15D and Hxk2S15A are nuclear excluded **even in glucose-grown cells**.

“Even in glucose-grown cells” doesn’t make sense to me because Hxk2 is normally nuclear excluded in glucose-grown cells.

Second, In two places in the ms. I’m told that Hxk2 forms dimers (and monomers) in high glucose, but forms predominantly monomers in low glucose:

Rigorous biochemical analyses demonstrate that in **glucose-replete conditions**, a

89 balance of **monomeric and dimeric** Hxk2 exists, but Hxk2 shifts to **predominantly**

90 **monomeric when glucose is restricted** [29–32].

In a **glucose-rich** medium, Hxk2 exists in a balance between **dimeric and**

210 **monomeric** species [32,33]. Upon **glucose starvation**, this balance shifts toward the

211 **monomeric** state [32,33].

But then I’m told that high glucose “destabilizes the dimer” and that “low glucose encourages dimerization”.

In **high glucose**

346 **concentrations**, bound glucose might disrupt N-terminal-tail binding within the catalytic

347 pocket to **destabilize the dimer** (Fig 4A). Alternatively, N-terminal-tail binding may

348 prevent glucose binding in **low glucose concentrations, encouraging dimerization.**

I like to offer authors suggestions for captivating titles (see <https://genestogenomes.org/how-to-write-titles-that-tempt/>). Here’s my suggestions for this paper:

Whither Hexokinase 2? To the nucleus when glucose is scarce, but not to regulate gene expression.

Glucose regulation of gene expression in S. cerevisiae: no role for Hexokinase 2.

No role in glucose regulation of gene expression for Hexokinase 2 of *S. cerevisiae*

Finally, the ms. is long. I suspect it could be modestly shortened (and made clearer) with aggressive editing. While editing the ms., I urge the authors to follow Strunk and White’s dictum: “avoid needless words.” Below are my suggestions of text that can be deleted. (I realize this will achieve only modest shortening, but I thought I should provide some examples. And these are only suggestions; I hope they’re useful.)

14 ~~In this study~~, we use**d** high-resolution, quantitative, fluorescent microscopy of live

98 localization, dimerization, and nuclear function of Hxk2. Our data ~~directly~~ contradict

~~105 Importantly, our imaging studies differ substantially from earlier work, which examined~~

~~106 fluorescently tagged Hxk2 localization using yeast cells that were likely experiencing~~

~~107 glucose starvation due to a pre-incubation in medium containing glycerol and lacking~~

~~108 glucose [8,11,13,14]. In contrast to these earlier studies, we maintain the indicated~~

~~109 glucose supply for all live-cell imaging, ensuring a more accurate representation of what~~

~~110 happens in cells under these conditions.~~

~~Future work will tackle this exciting aspect of Hxk2~~

~~125 biology, building off the new paradigm for Hxk2 nuclear translocation established herein.~~

122 together, our data ~~strongly~~ refute the idea that Hxk2 moonlights as a transcriptional

145 ~~To strengthen these observations,~~ we quantified for each hexokinase: (1) the

First, ~~as~~

167 ~~mentioned above~~, earlier studies incubated cells in glycerol or PBS (i.e., lacking

~~525 Surprisingly, the loss of Reg1 completely abolished Hxk2 nuclear translocation in~~

~~526 response to glucose starvation (S9B-D Fig). Thus, Reg1 — and likely, by extension, the~~

~~527 PP1 phosphatase, Glc7 — are required for Hxk2 nuclear accumulation in response to~~

~~528 glucose starvation. We will examine the impact of this regulator on Hxk2 in future~~

~~529 studies because we have not yet determined if Reg1 directly influences Hxk2~~

~~530 phosphorylation or indirectly regulates Snf1 or other Glc7-Reg1 substrates.~~

(I think this just confuses things. I suggest holding this back for another paper, once you know move about how Reg1/Glc7 is involved.)

531 ~~Elegant studies from Kriegel and Kettner demonstrate that~~ the Tda1 kinase is

597 ~~Earlier studies proposed that Hxk2 is a subunit of a DNA-bound repressor~~

598 ~~complex that regulates gene expression and, in particular, glucose repression~~ [7,15].

637 glucose conversion to G6P, the ~~critical~~ first step in glycolysis. In addition to cytosolic

661 ~~Here we report high-resolution, live-cell, confocal microscopy of fluorescently~~

662 ~~tagged Hxk2 performed in high (2% glucose) and low (0.05% glucose) glucose medium.~~

689 been openly questioned [23]. ~~These aspects, together with our findings, strongly~~

~~690 suggest that the previous model of Hxk2 nuclear accumulation in yeast is not correct.~~

769 the nucleus. However, in many instances, the nuclear function is poorly understood ~~both~~

770 ~~biochemically and mechanistically~~ [73]. Glucokinase (GCK) regulation by glucokinase

~~802 In future work, it will be critical to consider these existing models for hexokinase~~

~~803 and glucokinase nuclear regulation and function when examining the role of nuclear~~

~~804 Hxk2 in yeast. We hope this new paradigm for Hxk2 nuclear regulation and the powerful~~

~~805 genetic approaches available in yeast will help shed mechanistic light on the modes of~~

~~806 hexokinase and glucokinase function in humans.~~
